# Antibody-Dependent and Antibody-Independent Hemolysis in Sickle Cell Disease

**DOI:** 10.3390/antib15040070

**Published:** 2026-08-06

**Authors:** Raeshun T. Glover, Robert W. Maitta

**Affiliations:** Department of Pathology, University Hospitals Cleveland Medical Center, Case Western Reserve University, Cleveland, OH 44106, USA; raeshun.glover@uhhospitals.org

**Keywords:** sickle cell disease, antibody, autoantibody, alloantibody, red blood cell, hemolysis, hyperhemolysis, alloimmunization, heme

## Abstract

Sickle cell disease (SCD) represents one of the most complex hematological diseases leading to a lifetime of physiological crises characterized by chronic hemolysis, inflammation, hypoxia, and anemia with changes to body systems that result in patients having shorter lifespans. Transfusions, either simple or as part of red cell exchanges, are often needed to lower the hemoglobin S levels to minimize the possibility of sickling of red blood cells (RBCs) and provide patients with greater oxygen carrying capacity. However, hemolysis in SCD patients is a common finding/presentation of the disease, especially during acute crises. One of the ensuing complications of a life of transfusions is the development of alloantibodies to RBC antigens despite partial or extended matching. This is further complicated by formation of autoantibodies even in the setting of RBC matching, suggesting that a hyperactive immune response in these patients is primed to respond with formation of antibodies. In a sub-cohort of patients, no antibodies are detected despite extensive investigation, and the ensuing hemolysis requires minimizing exposure to RBC transfusions to avoid developing a greater hemolytic process. Instead, immunosuppression or monoclonals that target complement or cytokines shown to be involved in this type of hemolysis are necessary. In this context, this narrative review will present antibody-dependent and antibody-independent mechanisms of hemolysis in SCD, including therapeutic approaches that target specific areas of the immune response that are possibly involved in the destruction of RBCs.

## 1. Introduction

Sickle cell disease (SCD) is an orphan blood disorder that affects between 100,000 and 200,000 Americans [1,2]. Of these, 90% of SCD patients are African American, and 4% are Hispanic or Latino, with a crude SCD birth prevalence of 4.83 per 10,000 (1/2070) live births and 28.54 per 10,000 (1/350) among African Americans [3]. Worldwide, up to 300,000 infants are born annually with the disease [2]. In the US, children with SCD who survive into adulthood have on average a life expectancy of 20 years shorter than the rest of the population, with a higher proportion of patients dying when transitioning from pediatric to adult care settings [2]. SCD represents a hereditary autosomal recessive genetic disorder caused by a mutation in the *HBB* gene encoding for the β-globin chain, leading to formation of the abnormal sickle hemoglobin (HBS) tetramer, which is poorly soluble and polymerizes under low oxygen concentrations [4]. This is responsible for the severe clinical features of the disease characterized by acute episodes of pain, stroke, acute chest syndrome, priapism, and chronic organ damage as well as osteonecrosis, renal failure and chronic hemolytic anemia [5]. Due to the need for chronic transfusions during their lifetime, SCD patients are likely to develop iron overload and possibly develop known complications secondary to excess iron deposition in their tissues. This is important since these iron-associated tissue damages may not be reversed by red blood cell (RBC) exchanges [6].

Erythrocytes of sickle cell patients are fragile, stiff, prone to sticking together at low oxygen tension, susceptible to damage in circulation and are thus capable of occluding/obstructing the microvasculature [7]. Recurrent RBC membrane deformation though repeated cycles of sickling and unsickling disrupt membrane integrity, leading to hemolysis [4]. Thus, the repetitiveness of vaso-occlusive crises explains the lower life expectancy of SCD patients. In regard to therapy, allogeneic hematopoietic stem cell transplants have the potential of being curative, but they remain a limited option in SCD due to donor availability, with best results reported in pediatric patients who have a matched sibling donor [8]. Therefore, the goal of this narrative review is to describe the mechanisms by which hemolysis occurs in SCD patients that involve antibody formation and those that become active in the absence of antibody being generated.

### Disease Presentation

Pain crises and hemolysis are frequent complications experienced by SCD patients. Despite these representing a common feature of the disease, the degree of hemolysis experienced by patients and the mechanisms by which erythrocyte destruction happens in SCD result in the broad phenotype spectrum of disease seen in patients [9,10]. Exposure to RBC degradation products such as free hemoglobin and arginase 1 released from lysed cells as well as higher plasma concentrations of asymmetric dimethylarginine initiate cascades of events that drive endothelial dysfunction, leading to systemic vasculopathy [11,12]. In addition, released RBC membrane fragments and derived extracellular vesicles such as microvesicles (MVs)/microparticles (MPs) represent danger signals that further exacerbate systemic inflammation of the microvasculature and oxidative stress, both of which are involved in direct tissue injury and tissue hypoxia [13]. Mouse SCD models have indicated that these MPs induce severe endothelial cell injury through the upregulation of NFκB and HMOX1, resulting in enhanced RBC–platelet adhesion and platelet aggregation/activation [14,15]. Thus, these RBC-derived MPs as well as those derived from platelets, white blood cells, and endothelial cells in the plasma of SCD patients stimulate a higher degree of inflammation and oxidative stress in patients during crises [16]. The resulting endothelial defects in the setting of inflammation cause pulmonary hypertension, leg ulcers, chronic renal insufficiency, priapism, and ischemic strokes [17].

Reactive oxygen species (ROS) production in sickled RBCs is mediated enzymatically by NADPH oxidase, which is modulated by the protein kinase C and Rac GTPase, as well as intracellular Ca^2+^ signaling, and further increased by plasma levels of transforming growth factor β1 and endothelin-1 [18]. Nitric oxide (NO) deficits also play a major role in sickle cell disease pathophysiology. This is important since endothelial damage is disseminated by increased consumption of protective anticoagulants such as protein C and protein S as well as reduced NO [19,20]. NO is scavenged and inactivated by free hemoglobin in a deoxygenation reaction that oxidizes hemoglobin and generates methemoglobin, which is incapable of binding oxygen [20]. Furthermore, enhancement in ROS formation through hydroxyl radical synthesis via Fenton-like chemical reactions further damages endothelium [21]. In this way, ROS formation impairs blood flow and leads to impaired angiogenesis [22]. This oxidative stress is one of the central pathological insults that, over time, leads to organ damage associated with the disease. NO also binds soluble guanylate cyclase, which mediates conversion of GTP to cGMP, and this increase in cGMP lowers intracellular calcium and dephosphorylates myosin, thus relaxing vascular smooth muscle and causing vasodilatation [23,24].

Thus, hemolysis results in rapid consumption and decreased production of vasodilator NO, resulting in vaso-constriction and occlusion at the center of pain crises [25]. Regrettably, clinical trials seeking to increase NO bioavailability have shown this intervention to not affect the occurrence of acute pain crises [26]. The failure of these trials may be in part explained by recent information indicating that SCD patients, something also seen in mouse models, have a high baseline concentration of blood nitrite, which represents a major reservoir for NO synthesis [27]. The overall concentration of nitrite was found to be higher in blood of SCD patients compared to controls, a finding confirmed in BERK and Townes homozygous SCD mice. Thus, it appears that this concentration is a direct result of the disease, however, it remains to be determined if nitrite concentration could be used as a biomarker of disease state or impending crises. Of note is the fact that NO is also a potent neurotransmitter in the perception of pain that, depending on concentration, can mediate and amplify the hypersensitivity (hyperalgesia) exhibited in neuropathic pain and overall pain responses of SCD patients [28]. This allows for the distinction of two temporally defined types of pain in SCD crises, one pain which is acute in nature due to impaired oxygen delivery during vaso-occlusive crises with release of acute inflammatory mediators such as cytokines and another pain that is chronic in nature due to prolonged hyperalgesia and sensitization secondary to repeated crises episodes [29].

Free hemoglobin and heme also represent erythrocytic-damage-associated molecular pattern (eDAMP) mediators that stimulate innate immune responses, leading to endothelial changes characterized by inflammation and enhanced leukocyte adherence [30,31]. During hemolysis, eDAMPs bind to toll-like receptor 4 (TLR-4) on endothelial cells, triggering extensive endothelial inflammation, oxidative stress, and vaso-occlusion in a self-expanding cycle that involves different systems [32,33]. Heme may also activate the innate immune complement system and stimulate surrounding neutrophils to release neutrophil extracellular traps, expanding further the inflammatory cascade during a crisis event [21,34,35].

## 2. Antibody-Mediated Hemolysis

**Figure 1:**
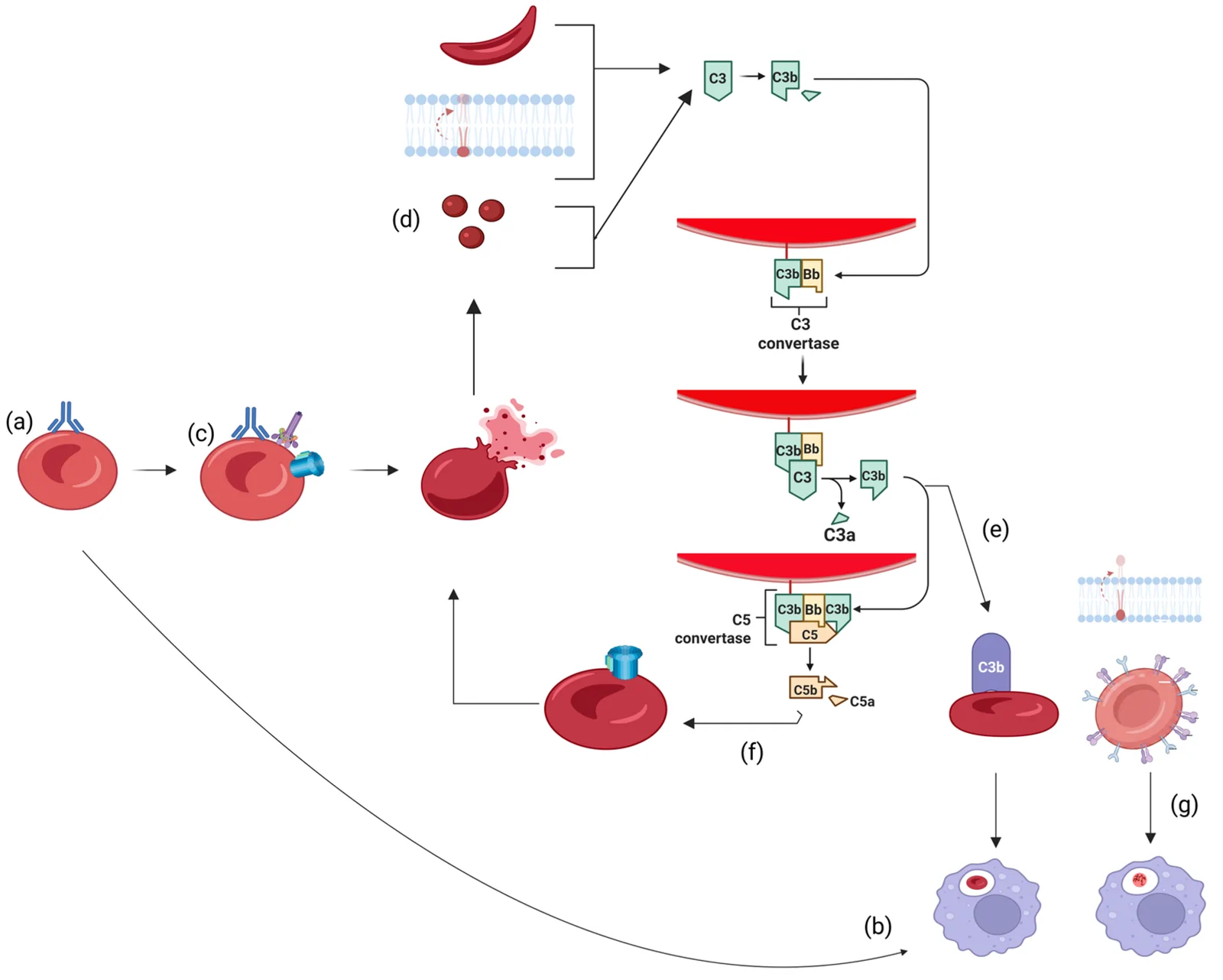
Pathogenesis of hyperhemolysis syndrome. *Antibody-mediated DHTR:* (**a**) Transfused incompatible RBCs are bound by recipient IgG alloantibody. (**b**) IgG-bound RBCs are phagocytosed by macrophages via Fc receptor undergoing extravascular hemolysis, representing a DHTR. (**c**) Alternatively, complement-activating IgG alloantibodies recruit C1q and lead to intravascular hemolysis of allogenic RBCs by the membrane attack complex (MAC) via the classical complement pathway. *Bystander hemolysis:* (**d**) Dense irreversible sickled cells in the setting of sickle crises, phosphatidylserine (PS) externalization due to deoxygenation and free heme released during prior intravascular hemolysis promote alternative complement pathway (AP) activation. (**e**) AP can terminate at C3b tagging allogenic and autologous RBCs for phagocytosis by macrophages via CR1. (**f**) AP can proceed to C5 convertase and MAC formation, leading to autologous and allogenic RBC intravascular hemolysis. *Reticulocytopenia:* (**g**) PS externalization and increased adhesion molecules including α4β1 and ICAM-4 on reticulocytes/mature RBC drive macrophage activation and phagocytosis, leading to reticulocytopenia and further extravascular hemolysis. Created in BioRender. Glover, R. (2026) https://BioRender.com/emx0sn5.

Hemolytic transfusion reactions due to antibody binding can be acute (AHTR) or occur much later after the transfusion event. In severe cases of delayed hemolytic transfusion reactions (DHTRs), hyperhemolysis often develops and a patient’s hemoglobin can decrease to concentrations lower than pre-transfusion levels. These DHTRs in SCD should not go unrecognized since they are potentially fatal, especially in the presence of “evanescent antibodies” [36,37]. These are antibodies that are present in blood under the detection testing thresholds that increase rapidly once a patient is re-exposed to the antigens eliciting the antibodies. In general terms, antibody-mediated hemolysis can be intravascular or extravascular depending on the complement-fixing ability of the IgG antibody. Complement-fixing IgG antibodies that activate the classical complement pathway and free hemoglobin, RBC MPs and hypoxia-altered endothelium mediate cell and tissue damage through activation of the alternative pathway [13,38]. When this occurs secondary to antibody binding, complement is activated through formation of the complement attack complex (*C5b*-9) (Figure 1) [38], which can lead to intravascular destruction of RBCs during the reaction, and favors the use of complement cascade blockers such as eculizumab in this setting [39]. Alternatively, non-complement-fixing IgG antibodies mediate extravascular hemolysis via phagocytosis of IgG-bound RBCs via Fc receptors by splenic macrophages (Figure 1). Thus, antibody formation as a result of transfusions is one of the most frequent complications of transfusion-based therapy in SCD patients. Notably, in the US differences, between a donor pool that is mostly Caucasian and SCD patients who are mostly African American is likely is one of the factors driving the high rate of RBC alloimmunization observed in the country [40].

When DHTR reactions occur due to antibody formation, SCD patients present with symptoms such as flank/back pain and dark urine from days to weeks after an RBC transfusion event. These would occur up to 14 days post the transfusion event, a time during which the evanescent antibody would increase and destroy the circulating transfused cells [41]. Thus, re-exposure to the previously sensitized RBC antigen via transfusion elicits immunological memory that triggers a secondary immune response that drives antibody production and subsequent destruction of transfused RBCs [36]. However, in up to a third of DHTR cases in SCD, no RBC alloantibodies can be identified [37]. Due to this, transfusion avoidance during DHTRs is strongly recommended, with long-term transfusion support becoming one of the factors taken under consideration when choosing a patient to undergo curative therapies such as stem cell transplantation or gene therapy if they have a history of DHTR [42].

### 2.1. Autoantibody Formation

Autoantibody formation is a common complication of chronically transfused SCD patients. Historically, it has been known that up to half of chronically transfused SCD patients develop warm autoantibodies during their lives [43,44]. However, formation of these autoantibodies is not limited to RBC antigens, since it has been reported that SCD patients form autoantibodies to other antigens such as rheumatoid factor (RF) and antiphospholipid antibodies [45,46], and these autoantibodies, when specific for a given antigen such as anti-nuclear, anti-DNA or anti-RF, increase the likelihood of developing overt autoimmune disease [47], which can affect as many as 10% of SCD patients [48,49]. Formation of these autoantibodies may be elicited in part due to the state of chronic inflammation affecting SCD patients’ microvasculature, exposure to RBC hemolysis byproducts such as cell-free heme, association with particular HLA antigenic determinants [50,51], and exposure to repeated blood transfusions since exposure to foreign antigens may prime the immune system to a hyperresponsive immunologic state [52].

In the setting of warm autoantibodies, red cell exposure needs to be avoided, since in most cases, all existing units will be incompatible upon crossmatching. This is especially the case when red cell exchanges have been performed, since they are ineffective and make the clinical situation worse due to the exposure to a greater number of RBC units during the procedures, accelerating red cell clearance, with a higher decrease in hemoglobin post-procedure [53]. Importantly, autoantibodies can be generated simultaneously with alloantibodies even after a single transfusion event [52]. Furthermore, even if extended antigen matching is performed for units given to SCD patients, the formation of autoantibodies is not affected [54]. It seems that greater antigenic exposure with a higher number of RBC units primes patients not only to make antibodies to minor antigens expressed by these cells but also to self-antigens leading to autoantibody formation [55]. Alternatively, the underlying chronic inflammation, tissue ischemia, and constant RBC breakdown in SCD can alter RBC membranes to expose cryptic antigens that trigger production of autoantibodies against them [56].

Paradoxically, it has been reported that cell-free heme is able to suppress both human B-cell plasmablast and plasma cell differentiation by inhibiting the DOCK8/STAT3 signaling pathway, which is essential for B-cell activation and antibody production, as well as upregulating heme oxygenase 1 (HO-1) through its enzymatic byproducts, carbon monoxide and biliverdin [57]. This suppression results in impaired humoral responses, dampened vaccination effectiveness and dysregulated alloimmunization responses during hemolytic events; however, this pathway appears to be inhibited in patients still not alloimmunized [57]. This data is significant, since it shows how hemolysis, regardless of triggering through heme, can have immune effects by reverting immune B cells to a more immature phenotype and a more non-specific/primitive antibody response. Nevertheless, the mechanisms by which autoantibody forms in SCD will require further investigation.

### 2.2. Alloantibody Formation

For the last couple of decades, there has been a greater effort to perform targeted matching for RHCE and Kell antigens in SCD patients, since historically antibodies to the RH antigenic group and Kell antigen have been reported to occur with greater frequency after transfusions [58]. This matching has allowed for a reduced incidence of alloimmunized in SCD patients, resulting in a decrease from 30–35% to as low as 3–14% [59]. However, just as in the case of autoantibody formation, exposure to a greater number of RBC units increases the likelihood of generating antibodies to antigens outside RH and Kell [60]. While RHCE and Kell matching is the current standard of care recommended by professional organizations such as the American Society of Hematology (ASH) [61], this does not eliminate the risk of alloimmunization. For instance, patients can still develop alloantibodies against other blood group systems, such as Duffy, Kidd, and MNS [62], even when blood is obtained from RHCE- and Kell-matched minority donors [63]. Nevertheless, the rate of alloimmunization has been reported to be lower using RBC units from minority donors due to a greater likelihood of antigenic similarities with recipients [40,64]. As a result, it has been proposed that genotyping patients is superior to phenotyping to detect variants specifically in RHCE expression [65]. However, obtaining a genotype may not translate into a reduction in alloimmunization, since in Brazil, even though genotype testing detected antigenic variants, this still did not result in elimination of alloimmunization [66]. To date, there are no trials comparing prophylactic genotype matching to serologic matching on alloimmunization rates [67]. Thus, judicious use of transfusions in SCD patients when possible is essential to minimize alloimmunization [60].

## 3. Hyperhemolysis Syndrome in Sickle Cell Disease

### 3.1. Hyperhemolysis Syndrome Clinical Presentation

Hyperhemolysis syndrome (HHS) is a rare but potentially fatal complication of transfusion. Approximately 13% of HHS cases are fatal [68]. While HHS can present in any patient with hematological conditions (myeloproliferative disorders, lymphomas, etc.), it predominately presents in patients with hemoglobinopathies such as sickle cell disease and beta-thalassemia [69,70]. In contrast to AHTR/DHTR, whereby the destruction of RBCs is limited to the transfused allogenic red cells, a defining characteristic of hyperhemolysis is the destruction of both autologous and allogenic RBCs (Table 1) [36].

While there are no formally accepted criteria for the diagnosis of HHS, clinicians have put forth diagnostic algorithms. One such diagnostic algorithm states that a diagnosis of HHS may be supported by the following criteria: (a) recent transfusion in the past 21 days, (b) a rapid decline in hemoglobin below the pre-transfusion baseline, (c) presence of hemolysis markers such as increased LDH, increased total bilirubin, increased plasma free hemoglobin and decreased haptoglobin, (d) reticulocytopenia, and (e) decreasing hemoglobin A (HBA)% in patients with sickle cell and/or beta-thalassemia on high-performance liquid chromatography (representing destruction of normal HBA transfused RBCs) [69]. These findings in part overlap with the features of hemolysis in the setting of DHTR or sickle cell crises as well. Thus, clinical findings alone are often insufficient to fully characterize the cause of hemolysis.

### 3.2. Hyperhemolysis Serological Evaluation

The serological evaluation for hemolytic transfusion reactions includes an indirect antiglobulin test (IAT), also known as an antibody screen, and a direct antiglobulin test (DAT). These tests are utilized to detect new or re-emerging alloantibodies that were not detected on pre-transfusion testing. The Centers for Disease Control and Prevention hemovigilance criteria states that for the diagnosis of a DHTR there must be an new alloantibody detected within the serum via the IAT or on the transfused RBCs via the DAT in association with hemolysis markers and recent transfusion [71]. When the serological findings of patients with hyperhemolysis were characterized, there was significant variability [72]. The IAT was positive in only 67.4% of cases, with only 39.2% of those demonstrating a new alloantibody. Only 55% of patients had a positive DAT, which demonstrated variable monospecific reactivity such as IgG (40%), C3 (36%) or both (24%) [72]. This variability in serological findings further delineates hyperhemolysis from the consistent serological findings of other hemolytic reactions such as AHTR and DHTR.

### 3.3. Antibody-Negative Hyperhemolysis

The significance of the large proportion of HHS cases without an alloantibody detected in either the IAT or DAT is debated. DAT detects antibody-/complement-coated RBCs that have escaped destruction. Therefore, a subset of these cases may have a negative DAT due to complete destruction of these RBCs in the setting of severe hemolysis [37]. Alternatively, the variability in the serological findings or the lack of a demonstrable alloantibody in some cases could highlight that underlying mechanisms of HHS may not be able to be only attributed to alloantibodies in all cases but through contribution of alternate mechanisms as well. However, it is recognized that the precipitating event often is alloantibody-mediated via DHTR. The predominating theory is that “bystander hemolysis” driven by complement activation and macrophage hyperactivation may also occur, which is responsible for the destruction of the autologous RBCs and reticulocytopenia [36,73] (Figure 1). Understanding the underlying multifactorial pathological mechanisms of hyperhemolysis is essential to diagnose and manage this complex entity.

### 3.4. Bystander Hemolysis Mediated by Complement Activation

As mentioned previously, SCD is associated with complement activation. Specifically, it has been shown that SCD patients have increased levels of Bb, C5a and soluble *C5b*-9, indicative of activation of the alternative complement pathway (AP) [74]. The increase in these levels correlates more with sickle cell crises than asymptomatic states [75,76]. The hyperactivation of the AP in SCD occurs through a variety of mechanisms. Firstly, a predominant site of activation is on a subset of sickled cells called dense irreversible sickled cells (DIRSCs) [9]. These cells bind C3 more readily and have a defect in surface-anchored complement regulators (CD35, CD55 and CD59) [74], which makes these cells potent initiators of dysregulated AP. Additionally, *C5b*-9 complexes have increased binding to DIRSCs, making them more susceptible to lysis [77,78]. Another mechanism includes membrane remodeling in sickled RBCs. Hemoglobinopathies can lead to dysregulation of membrane organization and maintenance. Notably, states of deoxygenation in SCD lead to the externalization of the normally inner-leaflet-based phosphatidylserine (PS) to the outer leaflet [79]. Likewise, studies have indicated that this leads to AP activation as well [80,81]. Lastly, that which may be of greatest significance to hyperhemolysis is AP activation due to free heme [82].

Free heme produced in the setting of RBC lysis during intravascular hemolysis can be partially scavenged by haptoglobin, preventing its deleterious effects; however, severe hemolysis often leads to its depletion and persistence of circulating free heme [83]. Besides its pro-inflammatory effects, free heme can cause hydrolysis of C3 and subsequent activation of the AP [13]. This activation of the AP leads to increased RBC lysis via *C5b*-9 with further free heme release. This results in a self-amplifying cycle of hemolysis [13,84]. Regarding hyperhemolysis, the cycle is often initiated by an alloantibody, causing destruction of transfused RBCs in a DHTR-like mechanism, causing the initial free heme release [36]. This initial DHTR-driving complement activation would explain the bystander hemolysis effect and the apparent paradoxical decrease in autologous RBCs.

Evidently, complement dysregulation is a key feature in SCD that may predispose these patients to HHS compared to the rarer cases of HHS in other diseases. Nevertheless, only a minority of SCD patients, approximately 5%, will ever have HHS [73]. This suggests that the pathophysiology of SCD alone may not be able to explain its occurrence but that there may also be genetic predisposing factors mediating HHS [85]. While not entirely clear which SCD patients may be predisposed to HHS, genetics studies have shown that mutations in complement-related genes such MBL2 (mannose-binding lectin 2) and KLCR3 (killer cell lectin-like receptor subfamily C, member 3) were associated with HHS cases [73]. Consequently, an association has been proposed between increased production of these ligands and an exaggerated membrane attack complex (MAC) response in these patients. Additionally, one study showed that mutations in genes such as thrombomodulin or ADAMTS13 may be a contributing factors [86]. Further genetic studies will be needed to clarify how well complement variants or complement-related genes contribute to predisposition of HHS due to complement activation.

### 3.5. Bystander Hemolysis Mediated by Macrophage Hyper-Activation

As previously discussed, SCD patients have increased hydrolysis of C3 to C3b and increase Bb levels, representing activation of the AP via mechanisms such as free heme or PS externalization [13,81]. If C3b complexes with Bb, then C5 convertase is formed and the pathway leads towards MAC formation and RBC lysis [87]. If, instead, factor I in the presence of Factor H leads to inactivated C3b (iC3b), MAC formation is prevented and RBCs are tagged for phagocytosis via CR1 receptors on macrophages [87,88]. Thus, macrophage activation is also a possible mechanism for HHS. Along these lines, patients with HHS have shown histopathological evidence of macrophage involvement. Erythrophagocytosis has been demonstrated on peripheral smears in these patients [89]. Moreover, presence of macrophages in the liver and spleen phagocytosing RBCs has also been described [90]. This confirms that extravascular hemolysis is a component of HHS.

Reticulocytopenia is a common finding in HHS, which contrasts to the expected reticulocytosis with other autoimmune hemolytic processes [91]. In other autoimmune hemolytic processes while RBCs are destroyed peripherally, the healthy marrow has a compensatory response, which drives increased RBC production and concordant reticulocytosis [92]. It has been proposed that PS externalization and increased adhesion molecules, including α4β1 and ICAM-4 on reticulocytes, tag them for peripheral clearance by macrophages [73]. However, in HHS, the marrow may be affected as well. Bone marrow evaluation in one case of HHS revealed diffuse marrow infiltration by macrophages with erythrophagocytosis and a background of erythroid hyperplasia [93]. Likewise, another more severe case demonstrated bone marrow necrosis [94].

### 3.6. Management of Hyperhemolysis

Once hyperhemolysis is suspected in a patient, transfusion medicine and hematology consultation is required to formulate a management plan [61]. Despite the low hemoglobin nadir of these patients, often below the threshold for transfusion, further RBC transfusion should be avoided. Further transfusions can “fuel the fire” by adding additional free heme from further hemolysis [95]. Transfusion ought to be limited to patients with severe hemodynamic instability. If transfusion is necessary, then the patient should receive full crossmatch-compatible, extended phenotype-matched units [61,69], preferably after administration of intravenous immunoglobulin (IVIG) and corticosteroids. ASH also recommends in their guidelines to give rituximab to prevent further alloimmunization [61].

**Figure 2:**
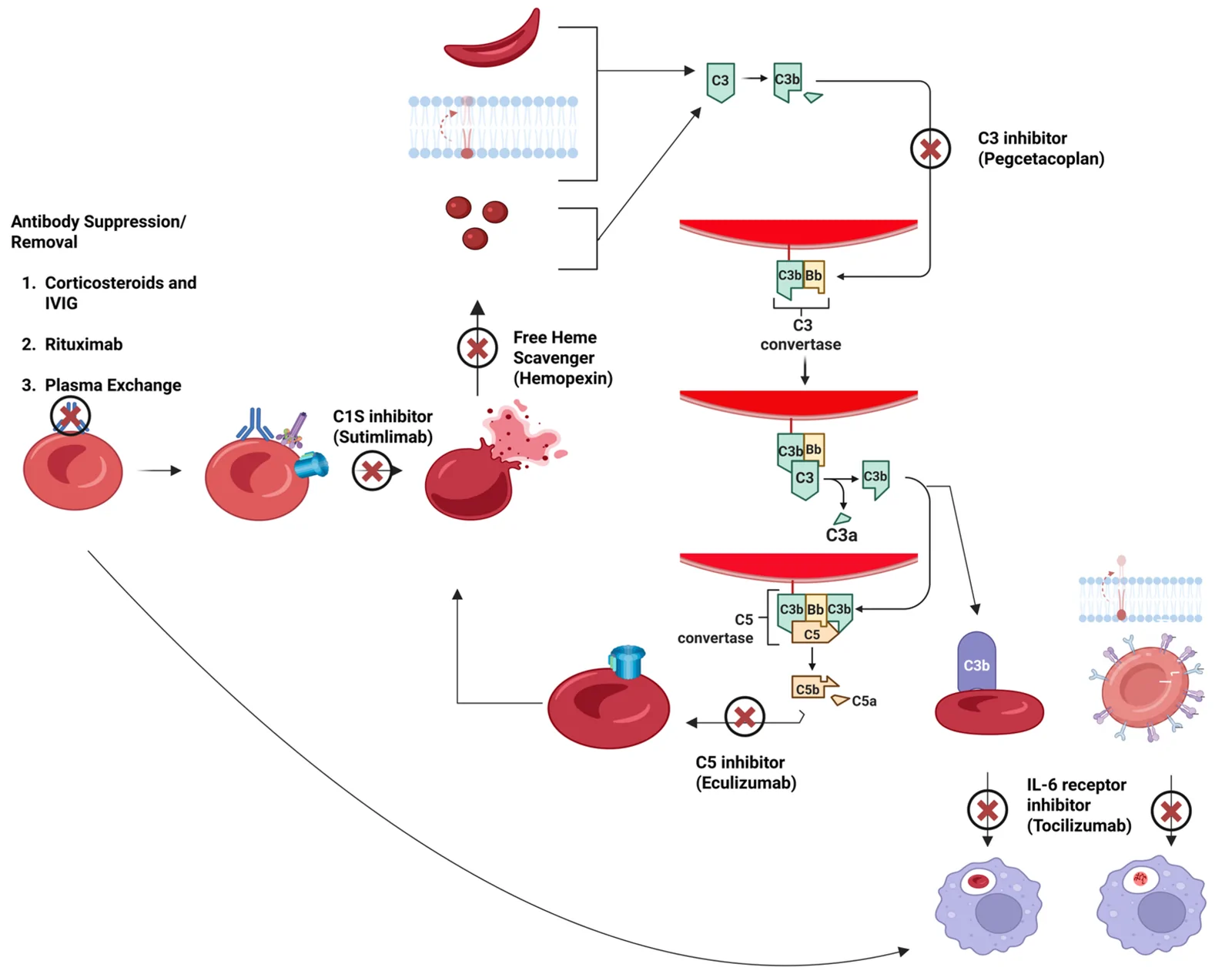
Current and theoretical therapeutic strategies in hyperhemolysis. Current first-line therapies include corticosteroids and IVIG. Additional treatment with eculizumab or tocilizumab may be considered. Rituximab is recommended in cases when supportive transfusion is needed to prevent further alloimmunization. As of yet, the effectiveness of more proximal complement therapies, including C3 inhibitors or C1s inhibitors, requires further investigation. The use of free heme scavengers such as hemopexin requires further investigation of efficacy as well. X on arrows represents steps targeted by therapies. Created in BioRender. Glover, R. (2026) https://BioRender.com/emx0sn5.

Given the variety of proposed underlying mechanisms for hyperhemolysis, there are an array of treatment modalities under investigation [69,72] (Figure 2 and Table 2). Management is primarily empirical with no clinical-trial-based evidence as of yet. An extensive workup should be undertaken to best determine the possible underlying mechanism(s) of HHS for guided treatment, as each of the therapies may have significant drawbacks. Firstly, immunosuppression of alloantibodies is targeted by giving the patient corticosteroids and IVIG [72]. These therapies are typically seen as first-in-line and are provided for most HHS patients [96]. Caution should be taken because of the risks of these therapies, as steroids can precipitate vaso-occlusive episodes in sickle cell patients [96]. Additionally, IVIG has a black box warning for hemolysis, a rare adverse event that may present in patients that are blood type A or AB [97]. Nevertheless, IVIG and corticosteroids may not target all pathways of HHS, and thus adjunct treatments targeting complement or macrophage activation may be needed as well [98].

The decision to use a complement inhibitor as part of HHS management varies by institution. Some may elect to give it to a patient regardless of the DAT results [99], while others may only elect to add it if the monospecific DAT for complement is positive [69]. DAT, however, may be falsely negative with severe intravascular hemolysis. For example, one study identified in DAT that negative cases of HHS increased levels of C3-positive RBCs by highly sensitive flow cytometry compared to healthy controls, further emphasizing that a C3-negative monospecific DAT may not entirely exclude complement involvement [68]. Alternatively, soluble *C5b*-9 may be an informative biomarker of complement activation as well and prove useful for tracking response to complement inhibitor therapy [84]. Eculizumab, a human monoclonal antibody and C5 inhibitor, is the predominant complement inhibition therapy used in HHS [100]. It prevents formation of the MAC (*C5b*-C9) responsible for intravascular hemolysis and decreases soluble sC5b9 levels [84,100]. However, eculizumab carries a black-box warning for risk of meningococcal disease, up to 2000-fold, even in the setting of vaccination [99]. Eculizumab does not target C3, and thus C3b still remains on RBCs, tagging them for extravascular hemolysis by macrophages [74,108]. Thus, more proximal complement cascade inhibitors are possible therapeutic options, such as C1s inhibitor (Sutimlimab) or C3 inhibitor (Pegcetacoplan) in HHS, which would not only prevent MAC formation but inhibit C3b formation responsible for phagocytosis by macrophages. These proximal complement inhibitors still remain to be investigated (Figure 2) [101,102].

Tocilizumab, an IL-6 inhibitor, may be added on to target the macrophage response [103]. Published cases where this therapy showed a measure of efficacy demonstrated that patients had significantly elevated ferritin levels and similar presentation to patients with macrophage activation syndrome [104]. It should be noted that SCD patients typically have iron overload at baseline from chronic transfusions, and thus the utility of iron level in diagnosing macrophage-mediated HHS requires further investigation [109]. Given that the cytokine response is a major driver of macrophage hyperactivation, a cytokine panel may be informative as well, but further investigation is needed to determine if HHS has a specific cytokine profile. One study used C-reactive protein (CRP), the downstream product of IL-6 stimulation, as a biomarker of HHS [103]. In some instances, clinicians have opted to use tocilizumab in patients with no demonstratable antibody, a systemic inflammatory clinical picture (i.e., fever and tachycardia), elevated inflammatory markers such as CRP and ferritin, or refractory to corticosteroids and IVIG [103,105]. Irrespectively, there is a gap in the best approach to elucidate if HHS is primarily macrophage-mediated to determine the need for tocilizumab.

Therapeutic plasma exchange (TPE) has been used in HHS patients with some effectiveness, but it is not typically utilized as it presents challenges [110,111]. Firstly, as these patients have severe anemia, an RBC prime would likely be required to prevent the hematocrit from decreasing further due to the apheresis procedure [112]. However, this prime has the potential to further exacerbate hemolysis. Secondly, the TPE procedure would remove many of the mainstay therapies from the patient, primarily the antibody-based therapies such as IVIG, eculizumab and tocilizumab, decreasing their therapeutic effectiveness [113,114]. As a result, the utility and timing of TPE in HHS patients remain to be determined.

HHS patients benefit from optimization of erythropoiesis. Iron, vitamin B12 and folate studies should be obtained, and deficiencies in each of these should be corrected [69,115]. Medications associated with marrow suppression such as hydroxyurea should also be discontinued during the presentation [105]. Similarly, an erythropoiesis-stimulating agent such as erythropoietin should be given [61]. Despite these approaches, recurrence of HHS can occur, but this is rare [110,111,116]. Patients with a history of HHS should receive RBC transfusions or red cell exchanges sparingly due to the risk of recurrence. Consequently, patients with a history of HHS should have flags within the blood bank laboratory information system and in the patient’s electronic medical record that would prevent them from getting unnecessary transfusions [98]. For patients with a history of HHS and transfusion requirements, pre-transfusion/post-transfusion prophylaxis with corticosteroids, IVIG, and rituximab should be explored in various combinations due to their possible therapeutic benefit [106,107].

## 4. Conclusions

This review has presented some of the known mechanisms by which hemolysis occurs in SCD. Formation of antibodies in a background of chronic inflammation caused by mediators released from lysed RBCs and via activation of immune responses that lead to formation of alloantibodies and at times autoantibodies to RBCs exemplify the complex mechanisms driving the short life expectancy of RBCs in patients having crises episodes or those who experience complications such as HHS. The latter is not an infrequent complication seen in SCD crises and represents a diagnostic challenge which at times requires an extensive investigation to rule out the presence of antibodies in the patient’s circulation. How complement, through either C3b or formation of MAC, leads to destruction of RBCs, including a patient’s own cells, exemplifies how difficult such cases are to treat. Roles for immunosuppression and monoclonals such as eculizumab and tocilizumab are presented in the context in which they may prove therapeutically useful. Finally, there needs to be further research into how complement and, in particular, how heme further expand the inflammatory microenvironment and how autoantibodies are still able to be generated despite extended RBC matching.

## Figures and Tables

**Table 1:** Delayed hemolytic transfusion reaction vs. hyperhemolysis syndrome.

	Delayed Hemolytic Transfusion Reaction (DHTR)	Hyperhemolysis Syndrome (HHS)
Definition	Immune-mediated destruction of allogeneic RBCs	Immune-mediated destruction of autologous and allogeneic RBCs
Mechanism	Alloantibody formation in the setting of an anamnestic immune response to antigen-positive RBCs	(1) Alloantibody formation in the setting of an anamnestic immune response to antigen-positive RBCs and/or (2) bystander hemolysis via complement activation and macrophage hyperactivation
Patient Population	History of transfusion, pregnancy or transplantation	Most commonly in sickle cell disease. Reported in association with thalassemia, myelodysplastic syndromes, and other hematological disorders
Onset	<28 days post-transfusion	Acute: <7 days of transfusion Delayed: 7–21 days post-transfusion
Post-Transfusion Hemoglobin	Remains at or above the pre-transfusion baseline with poor incrementation	Falls below the pre-transfusion baseline
DAT	IgG-positive with alloantibody in the eluate	Variable: DAT may be positive with IgG and/or C3d or entirely negative. A specific alloantibody can be eluted in a subset of cases
Antibody Screen	Positive with a new identified alloantibody	Variable: May be negative or positive with a new alloantibody
Transfusion	Provide antigen-negative units	Avoid transfusion unless severe hemodynamic instability

Sources: adapted from references [36,69,70,71,72,73].

**Table 2:** Current and theoretical therapeutic options for hyperhemolysis syndrome (Sources: Adapted from references [61,69,72,96,97,98,99,100,101,102,103,104,105,106,107].).

Therapeutic Agent	Mechanism of Action	Target Pathway	Use Context	Evidence Level	Serious Adverse Effects
Corticosteroids	Inhibits macrophage Fc receptor-mediated clearance of IgG coated RBCs	Antibody	First-line therapy	ASH-conditional recommendation (very low certainty)	Precipitation of vaso-occlusive crises in patients with SCD
Intravenous immunoglobulin (IVIG)	Inhibits macrophage Fcγ-mediated clearance of IgG-coated RBCs	Antibody	First-line therapy	ASH-conditional recommendation (very low certainty)	(1) Hemolysis from passive isohemagglutinins (particularly in blood-group-A recipients) (2) Thromboembolic events in patients with SCD
Rituximab	B-cell-directed therapy that reduces antibody production	Antibody	Indicated for prevention of further alloantibody formation in patients requiring transfusion	ASH-conditional recommendation (very low certainty)	Risk of infections. Especially in SCD with functional asplenia.
Eculizumab	Blocks cleavage of C5 preventing MAC (*C5b*-9) formation	Complement	Second-line therapy May be beneficial for complement-mediated HHS (DAT positive for complement may be informative)	ASH-conditional recommendation (very low certainty)	Risk of meningococcal/encapsulated bacterial infections
Sutimlimab/Pegcetacoplan	Inhibits proximal complement such as C1s or C3	Complement	Theoretically beneficial for complement-mediated HHS	Theoretical only Further investigation needed.	Risk of meningococcal/encapsulated bacterial infections
Tocilizumab	Blocks IL-6 receptor, inhibiting IL-6-directed macrophage activation	Macrophage	Given for refractory HHS May be beneficial for macrophage-hyperactivation-mediated HHS (elevated CRP and ferritin levels may be informative)	Limited evidence: case reports/series and systematic review (3 studies, total *n* = 20) [72,100,103].	Risk of developing serious infections
Hemopexin	Binds and scavenges free heme	Free heme	Theoretically beneficial for HHS with severe hemolysis (haptoglobin level may be beneficial)	Investigational drug product Phase I clinical trial ongoing	Not yet well established in clinical trials

## Data Availability

No new data were created or analyzed in this study. Data sharing is not applicable to this article.
